# 386. Participant Reflections on Their Experiences Participating in Phase 1 and 2 Inpatient Vaccine Trials and Human Challenge Studies

**DOI:** 10.1093/ofid/ofad500.456

**Published:** 2023-11-27

**Authors:** Bettina Wunderlich, Kathryn Chang, Lawrence Moulton, Nancy Kass, Ruth Karron, Kawsar R Talaat

**Affiliations:** Johns Hopkins University, Baltimore, Maryland; Johns Hopkins Bloomberg School of Public Health, Baltimore, Maryland; Pfizer Canada ULC, La Minerve, Quebec, Canada; Johns Hopkins University, Baltimore, Maryland; Johns Hopkins, Baltimore, Maryland; Johns Hopkins Bloomberg School of Public Health, Baltimore, Maryland

## Abstract

**Background:**

Volunteers are critical for the success of clinical trials but there is still debate on the ethics surrounding recruitment and enrollment of healthy human subjects who are paid for their participation in such research. This study seeks to examine the experiences and perceptions of risks and benefits of participants in early-phase inpatient studies to gain greater insight into how they feel about participating.

**Methods:**

An interviewer-administered survey was conducted with 152 healthy volunteers who participated in at least one Phase 1 or 2 inpatient vaccine or challenge clinical trial at the Johns Hopkins Center for Immunization Research, during the years of 2010-2020. Participant characteristics, study experiences, and perceived risks were analyzed. Poisson regression with robust variance was used to examine factors associated with perception of risk before and after participation.

**Results:**

Most participants who completed the survey were male (62%) and Black or African American (78%). 53% attained at most a high school degree and 42% had an income below $18,500 a year. Compensation was the primary factor for joining a study for 68% of participants; 35% of them relied on clinical trials as a major part of their income. Nevertheless, 32% reported they declined to participate in at least one study and 63% reported that they ask about the side effects before joining a new study. Over 90% of participants agreed or strongly agreed that they enjoyed participating, wanted to participate, and were glad they joined the study. There was a significant reduction in the perceived risk of study participation after study completion compared to perceived risk before joining the study, where before the study 46% thought it was “Not at all risky” compared to 73% afterwards.

**Conclusion:**

Most participants in our early-phase inpatient studies are males of lower income and compensation was the primary motivator for joining. However, side effects do factor in their decision whether to join. The majority of participants indicated that they were glad they joined the study. Despite the studies having risks and expected side effects, there was also a significant decline in perception of risks related to the study following participation, which implies participants feel safer than they anticipated feeling.

**Disclosures:**

**Lawrence Moulton, PhD**, Pfizer Canada ULC: Employee **Kawsar R. Talaat, MD**, Intralytix: Advisor/Consultant|Merck: Advisor/Consultant|NIAID: DSMB|Pfizer: Grant/Research Support|Pfizer: Pfizer contract with institution|Sanofi: Grant/Research Support|Takeda: Advisor/Consultant

